# Are men ready to use thermal male contraception? Acceptability in two French populations: New fathers and new providers

**DOI:** 10.1371/journal.pone.0195824

**Published:** 2018-05-29

**Authors:** Marie Amouroux, Roger Mieusset, Raoul Desbriere, Pierre Opinel, Gilles Karsenty, Marine Paci, Sara Fernandes, Blandine Courbiere, Jeanne Perrin

**Affiliations:** 1 Département Universitaire de Médecine Générale Faculté de Médecine de Marseille Aix-Marseille Université, Marseille, France; 2 Université Toulouse III-Paul Sabatier, Groupe de Recherche en Fertilité Humaine (Human Fertility Research Group), Toulouse, France; 3 Andrologie-Médecine de la Reproduction, Hôpital Paule de Viguier, CHU de Toulouse, Toulouse, France; 4 Service de Gynécologie-Obstétrique, Pôle Parents Enfants, Hôpital Saint-Joseph, Bd de Louvain, Marseille, France; 5 Centre Hospitalier Pays d'Aix, Aix-en-Provence, France; 6 Service d’Urologie et Transplantation Rénale, Aix Marseille Université, CHU La Conception, Marseille, France; 7 Aix Marseille Univ, INSERM, GMGF UMR_S 910, Marseille, France; 8 AP-HM La Conception, Centre Clinico-Biologique d'Assistance Médicale à la Procréation -CECOS, Marseille, France; 9 Laboratoire de Santé Publique, bd Jean Moulin–Marseille, France; 10 Aix Marseille Univ, CNRS, IRD, Avignon Univ, IMBE UMR 7263, Marseille, France; Population Council, UNITED STATES

## Abstract

**Background:**

Since the 1970s, international research has actively pursued hormonal male contraception (HMC) and, to a lesser extent, thermal male contraception (TMC). Although the efficacy of TMC has been confirmed in limited populations, its acceptability has not been studied in either potential users or potential prescribers.

**Methods:**

A cross-sectional descriptive multicentre study of potential male users of TMC (new fathers) and potential prescribers of TMC (new providers) was conducted between November 2016 and February 2017.The participants completed a 3-part survey, and their responses were evaluated to i) determine their socio-demographic profiles; ii) identify personal experiences with contraception; and iii) gauge the participants’ knowledge, interest and preference for male contraception, particularly TMC. For new providers only, the survey included a fourth part to evaluate professional experience with male contraception.

**Results:**

The participation rate was 51% for new fathers (305 NFs) and 34% for new providers (300 NPs, including 97 men (male new providers, MNPs) and 203 women (female new providers, FNPs)). Only 3% of NFs and 15% of NPs knew about TMC (including 26% of the MNPs and 10% of the FNPs, p<0.01). After reading information on TMC, new fathers were significantly less willing to try TMC (29%) than were new providers (40%) (p<0.01). The 3 main advantages of TMC for the new fathers included the following factors: “natural” (52%), “without side effects” (38%) and “non-hormonal” (36%). The main disadvantages were “lengthy wear time” (56%), “daily undergarment wear” (43%) and “concern about possible discomfort” (39%).

**Conclusions:**

Young male and female providers have limited knowledge of male contraception, are interested in further information and would generally prescribe TMC to their patients. Successful expansion of the use of male contraception, including TMC, would require distribution of better information to potential users and providers.

## Introduction

Recent studies have shown that among couples, women are the contraception users in more than 70% of cases [1]. Moreover, large-scale international studies have shown that most men are willing to assume responsibility for contraception by using male contraception [2–7].

Apart from vasectomy [8], the most well-known and most frequently used methods of male contraception include condoms or withdrawal [9]. Since the 1970s, international research has actively pursued male contraception, primarily hormonal approaches based on inhibiting gonadotropin secretion by administering exogenous androgen alone [10] or in combination with progestins [11] (for review, see [12,13]) and, to a lesser extent, thermal approaches. For both hormonal male contraception (HMC) and thermal male contraception (TMC), a first phase of 2 to 4 months consists of spermatogenesis inhibition to achieve a contraceptive threshold of less than 1 million sperm per mL of semen; once the threshold is obtained, treatments must be continued weekly (HMC, [10]) or daily (TMC) to maintain the contraceptive level [14].

TMC is based on the temperature dependence of spermatogenesis in most mammals, including men in whom testicular temperature is 2 to 5°C lower than core body temperature [15]. This lower physiological testis temperature results from two thermoregulatory systems: i) the testicular venous blood in vessels running on the surface of the testis loses heat through the scrotal skin—acting as a thermal exchanger between the inside and outside of the scrotal cavity—causing the testes temperature to fall to the same temperature as the subcutaneous tissue; and ii) precooling of the blood arriving to the testes by counter-current heat exchanges in the spermatic cord between the coiled spermatic artery—warm blood—running through the multiple veins of the anterior pampiniform plexus—cool blood—at the temperature of the subcutaneous tissue [16]. Among germ cells, pachytene and diplotene spermatocytes as well as spermatids are the most susceptible cells to increased testes temperatures; most of these affected cells undergo apoptosis when exposed to increased temperature [17], leading to a reduced sperm output depending on the temperature and/or exposure time.

Thermal approaches include induction of i) a high-intensity increase in scrotal temperature (38 to 46°C) for a short time once a day [18,19]; and ii) either +1°C in scrotal temperature during daytime hours [20] or +2°C in testis temperature for 15 or 24 hours daily [21–24] using the body as a source of heat to raise testis temperature [21–24]. These approaches result in a reduced sperm output [18–24]. In practice, using specific underwear (as shown in S1 File), each testicle is raised from the scrotum to the base of the penis near the external orifice of the inguinal canal, thus inducing a 2°C elevation in the testicular temperature.

The contraceptive efficacy of HMC was evaluated in two multicentre studies conducted by the World Health Organization [10] using androgen alone (testosterone enanthate by intramuscular (IM) injection once a week): 157 of the 271 men included became azoospermic and used this method as the only form of contraception with their partners over 1,486 months, with only one pregnancy noted, i.e., a Pearl index of 0.8 [10]. As azoospermia was not achieved in 42% of the men, a second study using the same treatment was performed in 357 men [20]; 80% of the men who achieved a sperm count within 0–3 million sperm/mL used this method as the only from of contraception with their partners, resulting in a Pearl index of 1.4, which is similar to a more recent study in Chinese men treated with monthly IM injections of testosterone undecanoate [25–27].

The contraceptive efficacy of TMC was evaluated as couples’ only form of contraception in three monocentre studies by inducing a 2°C increase in testicular temperature for 15 [28] or 24 hours/day [22,23]. Shafik reported no pregnancies in 28 couples over 252 months with an initial technique [22] and no pregnancies in 14 couples over 126 months with a second technique [22]; both techniques involved daily induction for 24 hours. In addition, no pregnancies occurred with a third technique involving daily induction for 15 hours/day in 9 couples over 158 months [28]. Therefore, the contraceptive efficacy of TMC was evaluated in a total of 51 couples over 536 months; no pregnancies were noted.

Although HMC acceptability has been evaluated in several studies [11,29,30], the acceptability of TMC has never been studied. Moreover, the influence of medical information on the choice of male contraception among physicians for their own use and their patients is poorly understood compared to other populations, such as female teenagers on long-lasting active contraception [31,32].

The primary objective of our study was to assess people’s knowledge regarding male contraception, including condoms, withdrawal, vasectomy, HMC and particularly TMC, and its hypothetical acceptability among two populations: new fathers and new providers of both sexes.

## Materials and methods

### Description of the study and the selected populations

We designed a multicentre, cross-sectional, descriptive study with two populations selected to represent potential future users and potential future prescribers of male contraception. The project was approved by the Ethical Committee of Aix Marseille University (number 2016-01-02-05).

The “new fathers” (NFs) group was designed to represent potential users and was composed of fathers recruited in maternity hospitals. We focused on men in stable relationships [4,6,9] and at a time in their lives (around childbirth) at which they may be questioning and experiencing concern regarding available contraception options. This population was called “new fathers” regardless of their ages and whether the men had become fathers for the first time.

The inclusion criteria were as follows: men whose female partners were hospitalized following childbirth and who agreed to participate in the study. No age limit was applied. The exclusion criteria were as follows: refusal to participate and lack of oral and written comprehension of the French language.

The “new providers” (NPs) group was designed to represent potential future prescribers of contraception and consisted of medical residents, “new” general practitioners and “new” obstetric or medical gynaecology practitioners (holding a Doctorate of Medicine for 2 years or less). These individuals were employed by the Public Assistance of the Hospitals of Marseille (AP-HM) and worked in hospitals located in the Marseille area or its close surroundings in the Bouches du Rhone Department. We targeted the specialties that most frequently manage contraception prescriptions. In addition, we selected interns or young physicians who had recently completed their training to provide the best possible evaluation of the initial training that these doctors currently receive regarding male contraception. The “new providers” population was therefore composed of two sub-populations: male new providers (population MNPs) and female new providers (population FNPs).

The inclusion criteria were as follows: women and men in an internship or employed as physicians for less than 2 years in the Bouches du Rhone Department who were willing to provide consent. No age limit was applied. The exclusion criteria were as follows: men and women in any other medical or surgical speciality who were employed as physicians for more than 2 years and were unwilling to participate in the study.

We selected two different populations for the study to explore the following variables:

- Differences in knowledge regarding male contraception and the acceptability of male contraception, particularly TMC, between new fathers and new providers;
- Hypothetical expectations of men regarding male contraception and providers’ interpretation of these expectations; and
- The influence of initial medical training on both the professional and personal attitudes of providers regarding male contraception, particularly TMC.

### Recruitment

To recruit the new fathers group, we selected 7 maternity hospitals from the 17 facilities (both public and private) in the Bouches du Rhone Department. Our selection was based on geographical categories and proximity to Marseille. Of the 7 institutions, only one declined to participate in our study, which was a private Catholic maternity centre.

This study was conducted in the following 6 centres: i) Centre Hospitalo-Universitaire (CHU, University hospital) La Conception Marseille, CHU Hôpital Nord Marseille, Centre Hospitalier du Pays d’Aix (CHPA) Aix-en-Provence Hospital and Aubagne Hospital (public facilities); ii) Hôpital Saint-Joseph Marseille (non-profit private hospital); and iii) Clinique Bouchard Marseille (private facility).

To recruit the new providers group, we sent requests through the Aix-Marseille University (AMU) mailing lists for residents and through the official mailing lists of the Assistants of AP-HM or Assistants Clinique Chiefs.

### Questionnaire

We developed an anonymous questionnaire for both populations. This form contained the following parts:

- The 1^st^ part, called “You”, consisted of 8 social and demographic questions;
- The 2^nd^ part, called “Contraception and you”, consisted of 6 questions for new fathers and 2 questions for new providers designed to evaluate the their personal experiences with contraception; 4 complementary questions designed for new fathers were related to contraceptive choices before and after pregnancy and how this choice was made;
- The 3^rd^ part, called “Male contraception”, consisted of 13 questions for new fathers and 11 questions for new providers; this part of the form contained an information page about TMC (see S1 File) and questions regarding knowledge, interests and preferences in terms of male contraception in general and TMC specifically after reading the information on the method;
- The 4^th^ part, called “You as a prescriber”, was intended only for new providers and consisted of 10 questions to evaluate the professional experiences of new providers regarding contraception.

For new providers, we intentionally asked the same questions regarding TMC twice: in the 3^rd^ part, the questions initially assessed the participants as users, and in the 4^th^ part, the same questions assessed their opinions as potential prescribers.

Overall, the questionnaire form for new fathers contained 3 different parts and 27 questions, the form for MNPs contained 4 parts and 33 questions, and the form for FNPs contained 4 parts and 31 questions. Answers were strictly anonymous, and only one answer was allowed per question. The time required to complete the questionnaire was less than 10 minutes. The forms are available in their entirety in S2–S4 Files.

### Data collection

For new fathers, the form was given directly to the fathers in the rooms of the hospitalized mothers in the presence of childbirth services; the form was given to the mother if the father was absent. An oral explanation was provided, written agreement was obtained, and then the completed forms were collected by either midwives or the administrator. We entered the answers into an Excel spreadsheet in Microsoft® Office 2010 (Microsoft Corporation, One Microsoft Way, Redmond, WA 98052 USA).

For new providers, the form was generated using "Google Form" software and was sent by email. Anonymous responses were saved automatically in the same software programme.

### Analysis and statistical tools

The socio-demographic characteristics of the participants who completed the form regarding the acceptability of TMC were analysed descriptively. Three distinct populations were described and analysed: new fathers, male new providers and female new providers. To define the social and professional categories, we used the 2008 ISCO-08 (International Standard Classification of Occupations).

In the description of the samples, qualitative variables were expressed as a percentage and the quantitative variables were summarized as the averages and standard deviations. The number of missing data was reported for each variable, and no imputation method was used.

For the quantitative variables, Student’s t-test for independent samples was used because the conditions for application were verified (normality of the distribution and homogeneity of the variances). For the qualitative variables, when the conditions for application were verified (theoretical number greater than or equal to 5), the Chi-squared test was used; otherwise, Fisher’s nonparametric test was used.

To determine the characteristics of people considering hypothetical use of male contraception, a group comparison was performed in both populations between those who answered “Yes” to this question and those who answered “No”.

All the statistical analyses were performed using IBM SPSS Statistics ver. 20.0 software.

### Ethics

We obtained approval from the Ethics Committee of Aix Marseille University (file number 2016-01-02-05).

## Results

All answers are presented in questionnaire for NF (S2 File), questionnaire for NMP (S3 File) and questionnaire for NFP (S4 File).

### Descriptions of the populations and contraception experiences

The following section lists the results from the 1^st^ part of the questionnaire: questions 1 to 14 for new fathers (8 social and demographic questions + 6 questions about “Contraception and you”, see S2–S4 Files), and questions 1 to 10 for new providers (8 social and demographic questions + 2 questions about “Contraception and you”, see S2–S4 Files).

Response rate: Between November 2016 and February 2017, we recruited 305 new fathers and 300 new providers (97 MNPs and 203 FNPs) for a total of 605 participants. The response rates were 50.8% for new fathers (305 of 600 distributed forms were completed) and 34.1% for new providers (300 of 879 sent forms were completed).

The gynaecologists’ (obstetricians and clinicians) response rate was 41.2%, and the general physicians’ response rate was 32.9% (p = 0.17).

Characteristics of the populations: The complete social and demographic characteristics of our three populations are presented in Table 1.

**Table 1:** Socio-demographic characteristics of the new fathers and the new providers.

N° question	Characteristics	New Fathers (NFs)	Male providers (MNPs)	Female providers (FNPs)	New providers (NPs)	p-value
	Effective	n = 305 (%)	n = 97 (%)	n = 203 (%)	n = 300 (%)	
Q1.1 Q2M1 Q2F1	**MEAN AGE** (years)	34.07 ± 6.02 a	27.65 ± 2.41	27.17 ±2.45	27.32±2,44 a	a: p<0.01
Q2M2 Q2F2	**MEDICAL SPECIALTY**					
	Medical Gynaecology	NA	0	5 (2.5)	5 (1.7)	
	Gynaecology-Obstetrics	NA	3 (3.1)	25 (12.3)	28 (9.3)	
	General medical practice	NA	94 (96.9)	173 (85.2)	267 (89)	
Q2M3 Q2F3	**WORKPLACE**					
	Private practice	NA	29 (29.9)	66 (32.5)	95 (31.7)	
	Medical Health centre	NA	4 (4.1)	11 (5.4)	15 (5)	
	Hospital	NA	64 (66)	126 (62.1)	190 (63.3)	
Q1.2	**EDUCATIONAL BACKGROUND**					
	No diploma	63 (20.7)	0	0	0	
	Baccalauréat diploma	46 (15.1)	0	0	0	
	1 to 5 years of studies	96 (31.5)	0	0	0	
	5 years or more	100 (32.8)	97 (100)	203 (100)	300 (100)	
Q1.4 Q2M4 Q2F4	**RELIGION**	106 (34.8) a	19 (19.6)	57 (28.1)	76 (25.3) a	a: p = 0.011
Q1.5 Q2M5 Q2F5	**IN A RELATIONSHIP**	304 (99.7) a	74 (76.3)	151 (74.4)	225 (75) a	a: p<0.01
Q1.6 Q2M6 Q2F6	**RELATIONSHIP DURATION** (years)	7.65 ± 4.32 a	3.87 ± 3.17	4.13 ± 3.20	4.04 ± 3.18 a	a: p<0.01
Q1.7 Q2M7 Q2F7	**NUMBER OF CHILDREN**	1.65 ±0.93 a	0.07 ± 0.30	0.13 ± 0.41	0.11 ± 0.38 a	a: p<0.01
Q1.8 Q2M8 Q2F8	**DESIRE FOR MORE CHILDREN (yes)**	198 (65.3)	82 (84.5)	177 (87.2)	259 (86.3)	

Column 1: The numbers of the questions in the questionnaire are presented in the S2–S4 Files) Q1: questionnaire for new fathers; Q2M: questionnaire for MNPs; Q2F: questionnaire for FNPs; Baccalauréat: French Diploma “A level”; NA: Not applicable; Significance threshold p<0.05; a:Comparison between new fathers and new providers; ± standard deviation.

- Among the new fathers (potential users), the median age was 33 years (min-max 19–56 years). The median duration of their relationships with their partners was 7 years (min-max 7–27 years). The median number of children per couple was 1 (min-max 0–6 children).
- Among the new providers (potential prescribers), the median age was 27 years (min-max 22–44). Most (267, 89%) were general practitioners, and 190 (63.3%) worked in hospital facilities. The median duration of their relationships with their partners was 3 years (min-max 0.04–20), and the median number of children was 0 (min-max 0–2).

The new fathers were significantly older, had more children and were more often in a stable relationship for a longer duration than the new providers.

Furthermore, the new fathers were more often practising a religion (p = 0.01). Because notation of religious denomination is not allowed in France, we could not discriminate the influence of different religions.

### Use of and experience with contraception

The complete results regarding the contraceptive experiences of the participants are available in Table 2.

**Table 2:** Contraceptive methods used among the new fathers and personal contraceptive experiences among the new fathers and new providers.

N° Question		New Fathers (NFs)			Male providers (MNPs)	Female providers (FNPs)	New providers (NPs)	p-value
Q1.11 Q1.14	**CONTRACEPTIVE METHODS**	**Before pregnancy**	**After pregnancy**					
	(multiple answers possible)	n = 298 (%)	n = 241(%)	-		-	-	
	Oral Contraception (Pill)	154 (51)	110 (45.6)	-		-	-	
	IUD	29 (9.7)a	73 (30.3)a	-		-	-	a : p<0.01
	Implant	6 (0.2)	10 (4.1)	-		-	-	
	Rhythm method of familly planning	0	1 (0.4)	-		-	-	
	Female condom	1 (0.3)	3 (1.2)	-		-	-	
	Cervical cap	0	0	-		-	-	
	Male condom	91 (30.5)	60 (24.9)	-		-	-	
	Withdrawal	40 (13.4)	12 (5)	-		-	-	
	HMC	0	1 (0.4)	-		-	-	
	TMC	0	1 (0.4)	-		-	-	
	None	51 (17.1)	2 (0.8)	-		-	-	
	Others	17 (5.7)	21 (8.7)	-		-	-	
Q1.12	**WHO MADE THAT CHOICE ?**	n = 300 (%)						
	Me	10 (3.3)	-	-		-	-	
	My partner (her)	87 (28.5)	-	-		-	-	
	Both	203 (67.7)	-	-		-	-	
Q1.9 Q2M9 Q2F9	**SIDE EFFECTS DUE TO CONTRACEPTION**	n = 301 (%)		n = 97(%)		n = 203(%)	n = 300 (%)	
	Yes for me	4 (1.3)	-	2 (2.1)		108 (53.2)	110 (36.7)	
	Yes for my partner (her)	65 (21.6)	-	50 (51.5)		NA	50 (16.7)	
	Yes for my partner (him)	NA	-	NA		7 (3.4)	7 (2.3)	
	Neither of us	232 (77.1)a	-	45 (46.4)		88 (43.3)	133 (44.3)a	a: p<0.01
Q1.10 Q2M10 Q2F10	**PREVIOUS UNWANTED PREGNANCY WHILE ON BIRTH CONTROL**	n = 301	-	n = 97		n = 203	n = 300	
	YES	38 (12.6)a	-	13 (13.4)b		11 (5.4)b	24 (8)a	b: p = 0.017 a: p<0.01

The numbers of the questions in the questionnaire are presented in S2–S4 Files; Q1: questionnaire for new fathers; Q2M: questionnaire for MNPs; Q2F: questionnaire for FNPs; n = effective; IUD: Intra uterine device; Implant: Subcutaneous implant; HMC: Hormonal Male Contraception; TMC: Thermal male contraception; Others: Nuvaring®, Décapeptyl®, Billings Method, Hypofertility, Monitoring of Ovulation, Unspecified; p<0.05 significance threshold; a:Comparison between NFs and NPs; b: Comparison between MNPs and FNPs; NS: Not significant; NA: Not applicable.

Among the new fathers, contraception before pregnancy was primarily ensured by the female partner via contraceptive pills (51%) and by the male partner via condoms (30.4%) and withdrawal (13.4%). After pregnancy, contraception was ensured primarily by the female partner (43% pills, 29% IUD) and secondarily by the male partner (24% condoms). Contraceptive choices were made by both members of the couple in 203 couples (67.7%).

Regarding personal experiences with contraception use, fewer side effects related to contraception were reported among the new fathers than among the new providers (p < 0.01).

Contraceptive failure occurred for 38 new fathers (12.6%), 13 MNPs (13.3%) and 11 FNPs (5.4%).

New providers reported more side effects of contraception than did new fathers. FNPs were significantly less likely to experience contraceptive failure than were new fathers and MNPs.

### Knowledge and use of male contraception

The following section presents the results of the 2^nd^ part of the form: questions 15 to 19 (5 questions exploring knowledge of male contraception, see S2–S4 Files) and questions 25 to 27 (“Would you like more information about male contraception in general?”, “Would you like a larger variety of choices for male contraception?”, and “Are you interested in having a vasectomy?”) for new fathers, questions 11 to 15 for MNPs and FNPs (5 questions exploring knowledge of male contraception, see S2–S4 Files), questions 21, 32 and 33 for MNPs and questions 11 to 15 and 20, 30 and 31 for FNPs (“Would you like a larger variety of choices for male contraception?”, “Would you like more information about male contraception in general?”, and “Would you be interested in participating to a training course about male contraception?”). We focused on the knowledge of the two populations regarding male contraception in general and on hypothetical use of a male contraceptive method by the couples.

All the results regarding male contraception are presented in Table 3.

**Table 3:** Male contraception: Known and preferred methods, acceptability and willingness among the new fathers and new providers.

N° Question	MALE CONTRACEPTION	New Fathers (NFs)	Male providers (MNPs)	Female providers (FNPs)	New providers (NPs)	p-value
Q1.15 Q2M11 Q2F11	**KNOWN MALE CONTRACEPTION**	n = 304 (%)	n = 97 (%)	n = 203 (%)	n = 300 (%)	
	Condom	298 (97.7)	96 (99)	202 (99.5)	298 (99.3)	NS
	Withdrawal	193 (63.3)	63 (64.9)	148 (72.9)	211 (70.3)	NS
	Vasectomy	146 (47.9)a	83 (85.6)	181 (89.2)	264 (88)a	a : p< 0.01
	HMC	34 (11.3)a	31 (32)	43 (21.2)	74 (24.7)a	a : p< 0.01
	TMC	8 (2.6)a	25 (25.8)b	20 (9.9)b	45 (15)a	a,b: p<0.01
	None	7 (2.3)	0	1 (0.5)	1 (0.3)	
	Other	4 (1.3)	4 (4.1)	6 (3)	10 (3.3)	
Q1.20 Q2M16 Q2F16	**PREFERRED METHOD after receiving information**	n = 303 (%)	n = 97 (%)	n = 203 (%)	n = 300 (%)	
	Condom	196 (64.5)	71 (73.2)	130 (64)	201 (67)	
	Withdrawal	49 (16.1)a	3 (3.1)	10 (4.9)	13 (4.3)a	a : p<0.01
	Vasectomy	8 (2.6)	3 (3.1)	4 (2)	7 (2.3)	
	HMC	5 (1.6)a	2 (2.1)	16 (7.9)	18 (6)a	a : p = 0.04
	TMC	30 (9.9)	12 (12.4)	27 (13.3)	39 (13)	NS
	None	18 (5.9)	5 (5.2)	15 (7.4)	20 (6.7)	
	Other	6 (2)	1 (1)	1 (0.5)	2 (0.7)	
Q1.16 Q2M12 Q2F12	**WOULD YOU AGREE TO USE MALE CONTRACEPTION?**					
	**YES**	178 (58.4)ac	68 (70.1)bc	150 (73.9)bc	218 (72.7) a	a,c : p<0,01 b : NS
Q1.17 Q2M13 Q2F13	**Main reason**	n = 178 (%)	n = 68 (%)	n = 150 (%)	n = 218 (%)	
	To share responsibility	91 (51.4)	41 (60.3)	90 (60.4)	131 (60.4)	
	To have an extra safety measure to avoid pregnancy	27 (15.3)	3 (4.4)	17 (11,4)	20 (9.2)	
	To avoid having a child unknowingly	14 (7.9)	7 (10.3)	NA	7 (10.3)	
	To avoid side effects due to female contraception	67 (37.9)a	16 (23.5)	41 (27.5)	57 (26.3)a	a : p = 0.014
	To avoid the risk of having a child with another partner	4 (2.3)	0	NA	0	
	To prevent my partner from having a child with another partner	NA	NA	0	0	
	Other	1 (0.6)	1 (1.5)	2 (1.3)	3 (1.4)	
	**NO**	127 (41.6)	29 (29.9)	53 (26.1)	82 (27.3)	
Q1.18 Q2M14 Q2F14	**Main reason**	n = 127 (%)	n = 29 (%)	n = 53 (%)	n = 82 (%)	
	Inconvenient	27 (21.6)	9 (31)	NA	9 (31)	
	Side effects	18 (14.4)	9 (31)	NA	9 (31)	
	Contraception belongs to women	16 (12.7)	3 (10.3)	4 (7.5)	7 (11.3)	
	Damage to virility	9 (7.2)	2 (6.9)	6 (11.3)	8 (12.9)	NS
	Not interested at all	49 (39.2)	3 (10.3)	12 (22.6)	15 (24.2)	
	Other	23 (18.4)	3 (10.5)	16 (30.2)	14 (22.6)	
	I do not trust my partner	NA	NA	15 (28.3)	NA	
Q1.25 Q2M32 Q2F30	**DESIRE FOR MORE INFORMATION**	n = 301 (%)	n = 97 (%)	n = 203 (%)	n = 300 (%)	
	YES	130 (43.2)ac	81 (83.5)c	195 (96.1)c	276 (92)a	a,c : p<0.01
Q1.26 Q2M21 Q2F20	**WILLINGNESS TO HAVE MORE CHOICES**	n = 301 (%)	n = 97	n = 203 (%)	n = 300 (%)	
	YES	163 (54.2)ac	72 (74.2)c	182 (89.7)c	254 (84.7)a	a,c : p<0.01

The numbers of the questions in the questionnaire are presented in S2–S4 Files; Q1: questionnaire for new fathers; Q2M: questionnaire for MNPs; Q2F: questionnaire for FNPs; n = effective; HMC: Hormonal male contraception; TMC: Thermal male contraception; p<0.05 significance threshold; a: Comparison between NFs and NPs; b: Comparison between MNPs and FNPs; c: Comparison between NFs and MNPs (men) and FNPs (women); NS: Not significant; NA: Not applicable.

Knowledge regarding male contraception was homogeneous among both populations in terms of condom use and withdrawal, which were known in 98 to 99% of the subjects.

Regarding vasectomy, the new providers were significantly better informed than the new fathers (88% versus 48%, respectively).

The new providers were more familiar with the so-called “new” contraceptive methods (HMC and TMC) than the new fathers (p<0.01). The MNPs were more aware of TMC than the FNPs (26% versus 10%, p<0.01). Nevertheless, these methods were not widely known to either population.

After receiving information regarding TMC, among all the suggested methods, the favourite methods of the participants were as follows:

- New fathers: condoms (64.5%), followed by withdrawal (16.1%) and TMC.
- New providers: condoms (67%), followed by TMC, which was the second favourite method for 39 physicians (13%). This method was ranked higher than HMC, vasectomy and withdrawal.

The following results were observed for the question “Would you be ready to use male contraception as your couple’s form of contraception?”:

- 58.4% of the new fathers responded “Yes” versus 70.1% of the MNPs and 73.9% of the FNPs. A statistically significant difference was observed between the new fathers and the new providers (p < 0.01). The main motivations were as follows (Table 3):
  - share the contraceptive responsibility (51.4% of new fathers versus 60.4% of new providers); and
  - avoid side effects related to female contraception (37.9% of new fathers versus 26.3% of new providers).
- When interviewing the 53 female providers (FNPs) who answered “No” regarding their future use of male contraception, the main reason was a lack of confidence in the partner (28.3%). Damage to virility was not a major concern, although it was more frequently emphasized by the FNPs than by the men (NFs and MNPs), with no significant difference. For the male populations (NFs and MNPs), the main reasons for responding “No” to future male contraception use were a hypothetical “overly restrictive feeling” (21.6% and 31%, respectively) and a hypothetical “fear of side effects” (14.4% and 31%, respectively).

Statistically, more new fathers who belonged to higher social and professional categories or had a higher education level were part of the group that answered “Yes, I am ready to ensure contraception by using male contraception” than those who answered “No”. Therefore, a statistical link was observed within the new fathers between the subjects interested in male contraception and those with a strong educational level (69% had a master’s degree in the group “Yes” versus 31% in the group “No”; p = 0.07). In addition, 70.5% of the subjects with intellectual and scientific professions (2 on the ISCO-08 classification) were willing to use male contraception in the group “Yes” versus 29.5% in the group “No” (p = 0.046).

Experiences with contraception also influenced the attitudes of the new fathers (75%, p = 0.028) and FNPs (82.4%, p = 0.008), and a statistically greater number of new fathers and FNPs experienced contraceptive side effects in the “Yes, I am willing to use male contraception with my partner” group than in the “No” group.

Among the new providers, religion influenced the attitudes of the men: a statistically greater number of men who answered “No” for the hypothetical use of male contraception (52.6%) practised a religion than the men who answered “Yes” (47.4%, p = 0.016).

Finally, both studied populations wanted more information and more choices for male contraception, with more information requested by 43.2% of the new fathers and 92% of the new providers, and more choices requested by 54.2% of the new fathers and 84.6% of the new providers.

### User attitudes towards thermal male contraception

The following section presents the results of the 3^rd^ part of the questionnaire after the information page about TMC, which included questions 20 to 24 for new fathers (the type of male contraception that they would be willing to use, advantages and disadvantages of TMC, willingness to try TMC and time in a man’s life that would be most appropriate for TMC use, see the S2–S4 Files). The aim was to assess the expectations and fears of potential users (NFs) regarding TMC. All the results concerning TMC among the new fathers are presented in Table 4.

**Table 4:** Knowledge and hypothetical acceptability of thermal male contraception among the new fathers.

N° Question	THERMAL MALE CONTRACEPTION	New fathers	
Q1.19	**HAVE YOU EVER HEARD ABOUT TMC?**	n = 305 (%)	
	YES		17 (5.6)
Q1.21	**ADVANTAGES OF TMC**		n = 303 (%)
	Environmental		87 (28.6)
	Inexpensive		61 (20.1)
	No adverse effects		117 (38.5)
	Efficiency		60 (19.7)
	Non-Hormonal		110 (36.2)
	Natural		158 (5)
	Reversible		109 (35.9)
	Other		27 (8.9)
	None		NA
Q1.22	**DISADVANTAGES OF TMC**		n = 303(%)
	Delayed effectiveness		94 (30.9)
	Delayed reversibility		64 (21.1)
	Time required for wear		170 (55.9)
	Aesthetic		67 (22)
	Uncomfortable		118 (38.8)
	Must be worn without fail		131 (43.1)
	Loss of confidence		103 (33.9)
	Damage to virility		41 (13.5)
	STI risk		51 (16.8)
	Other		27 (8.9)
Q1.23	**WOULD YOU ACCEPT TO TRY?**		n = 304 (%)
	I woud totally accept		22 (7.2)
	I would generally accept		67 (22)
	I would generally not accept		92 (30.3)
	Not at all		123 (40.5)
Q1.24	**WHICH PERIOD OF A MAN’S LIFE WOULD BE THE MOST APPROPRIATE?**		n = 89 (%)
	Single		12 (4)
	Unstable relationship		8 (2.7)
	Before having the first child		17 (5.6)
	Between two children		40 (13.3)
	After children’s birth		64 (21.3)
	If female contraception is impossible		59 (19.6)
	No opinion		99 (32.9)
	Other		16 (5.3)

The numbers of the questions in the questionnaire are presented in S2 File; Q1: questionnaire for new fathers; n = effective; TMC: Thermal male contraception; NA: Not applicable.

First, we determined whether the men interviewed had already heard of this method, which enabled us to evaluate general knowledge regarding TMC.

After providing an information page on TMC, including its strengths and weaknesses, hypothetical use of this method was assessed. Overall, 29.2% of the new fathers were in favour of trying TMC, and they found that its main benefits included being natural (52%), non-hormonal (36.2%) and without side effects (38.5%). Among all new fathers, the major disadvantage mentioned was the length of use, which was too long (55.9%), followed by daily wearing of underwear (43.1%) and fear of discomfort (38.8%).

We asked the participants about the most appropriate time in one’s life to try TMC. Among the new fathers interested in TMC, 40.2% believed that the best time would be when they were in a stable relationship (before, between or after children). Within this sample, 21.3% were more likely to try TMC after having children.

Among the new fathers, no significant differences were observed between those who would “totally agree”or “generally agree” to try TMC and those who would “rather not agree” or “not agree at all”for the following characteristics: education level, religious practice (29.2% versus 37.2% respectively, p = 0.19), history of contraception failure (8.9% versus 14.2%, respectively, p = 0.26), type of contraception used before pregnancy and who chose the type of contraception in the couple. Conversely, among the new fathers who would agree to try TMC, we observed a significantly higher proportion of men who had experienced contraception side effects (31.3% versus 18.6% in the new fathers who would not agree to try TMC, respectively, p<0.05) and who would be ready to undergo vasectomy (5.6% versus 1.4%, respectively, p<0.05).

Among the male new providers, no significant differences were observed between those who would “totally agree” or “generally agree” to try TMC and those who would “rather not agree” or “not agree at all” for the following characteristics: type of medical practice (private or public), religious practice (16.7% versus 20.9%, respectively, p = 0.63), history of contraception failure (16.7% versus 11.9%, respectively, p = 0.53), being in a relationship or not (80% versus 74.6%, respectively, p = 0.56) and willingness to have children or not (90% versus 82.1%, respectively, p = 0.38). Conversely, among the male new providers who would agree to try TMC, we observed a significantly higher proportion of men who had experienced contraception side effects (70.0% versus 46.3% in the male new providers who would not agree to try TMC, p<0.05).

### Prescriber attitudes towards thermal male contraception

The next section presents the 4^th^ part of the form (only new providers were given this part to analyse them as prescribers of contraception), which included questions 23 to 33 (MNPs) and 21 to 31 (FNPs): frequency of prescription contraception requests; feelings regarding the current means of contraception; frequency and type of male contraception prescribed; reasons for not prescribing vasectomy, HMC or TMC; willingness to recommend male contraception by hyperthermia; advantages and disadvantages of TMC for patients; desire for more information about male contraception in general and interest in participating in a training course about male contraception (see S2–S4 Files).

We asked the new providers (potential future prescribers) about male contraception to identify their professional attitudes towards contraception in general and then their personal experiences with male contraception. Finally, we ascertained their opinions on TMC.

All results regarding professional experiences with male contraception among the new providers are listed in Table 5.

**Table 5:** Frequency of consultation for family planning and attitudes of the new providers towards male contraception as prescribers.

N° Question		Male providers (MNPs)	Female providers (FNPs)	New providers (NPs)	p-value
**Q2M23 Q2F21**	**HOW OFTEN ARE YOU ASKED FOR CONTRACEPTION?**	n = 97	n = 203	n = 300	
	Very often- Often	40 (41.2)b	116 (57.1)b	156 (52)	b : p <0.01
	Rarely–Very rarely	43(44.3)b	61 (30)b	104 (34.7)	b : p = 0.01
	Never	14 (14.4)b	26 (12.8)b	40 (19.7)	b : NS
**Q2M24 Q2F22**	**DO YOU FEEL UNEQUIPPED REGARDINNG CURRENT MEANS OF CONTRACEPTION?**	n = 97	n = 203	n = 300	
	Yes	43 (44.3)b	110 (54.2)b	153 (51)	b : NS
	No	54 (55.7)	93 (45.8)	147 (49)	
**Q2M25 Q2F23**	**HOW OFTEN DO YOU OFFER MC?**	n = 97	n = 203	n = 300	
	Very Often- Often	16 (16.4)b	36 (17.7)b	52 (17.3)	b : NS
	Rarely–Very rarely	44 (45.3)b	103 (50.7)b	147 (49)	b : NS
	Never	37 (38.1)b	64 (31.5)b	101 (33.7)	b : NS
**Q2M26 Q2F24**	**HAVE YOU EVER OFFERED OTHER TYPES OF MC BESIDES CONDOMS?**	n = 97	n = 203	n = 300	
	Very Often–Often	6 (6.2)b	3 (1.5)b	7 (2.3)	b : p = 0.02
	Rarely–Very rarely	40 (41.2)b	95 (46.8)b	135 (45)	b : NS
	Never	51 (52.6)b	105 (51.7)b	156 (52%)	b : NS
**Q2M33 Q2F31**	**WOULD YOU BE INTERESTED IN PARTICIPATING TO A TRAINING COURSE ABOUT MC?**	n = 97	n = 203	n = 300	
	Yes	74 (76.3)b	173 (85.2)b	247 (82.3)	b : NS
	No	23 (23.7)	30 (14.8)	53 (17.7)	
**Q2M28 Q2F25**	**OTHER MC THAN CONDOM OFFERED**	n = 97	n = 203	n = 300	
	Vasectomy	37 (38.5)b	78 (38.4)b	115 (38.3)	b : NS
	HMC	5 (5.2)b	9 (4.4)b	14 (4.7)	b : NS
	TMC	3 (3.1)b	3 (1.5)b	6 (2)	b : NS
	None	55 (57.3)b	118 (58.1)b	173 (57.7)	b : NS
	Other	2 (2.1)b	5 (2.5)b	7 (2.3)	b : NS

The numbers of the questions in the questionnaire are presented in S3 and S4 Files; Q2M: questionnaire for MNPs; Q2F: questionnaire for FNPs; n = effective; MC: Male contraception; HMC: Hormonal male contraception; TMC: Thermal male contraception; p <0.05 significance threshold; a: Comparison between NFs and NPs; b: Comparison between MNPs and FNPs; c: Comparison between NFs and MNPs (men) and FNPs (women); NS: Not significant; NA: Not applicable.

First, we noted homogeneity in the responses between the MNPs and FNPs (no significant differences between their responses), particularly regarding their infrequent prescribing of male contraception. Indeed, one-third of the new providers (33.7%) never offered male contraception during a contraceptive consultation.

In addition to condoms, the doctors mainly proposed vasectomy but felt that they lacked referral physicians or sufficient training. The main reason that they did not propose male contraception more often was because they did not consider it during consultation.

More than half of the participants never mentioned methods other than condoms (156 people, 52%). Indeed, HMC and TMC were not offered because the individuals were not aware of these methods (Q2M29 a-b-c, S3 File, Q2F26 a-b-c, S4 File).

However, a significant difference was observed between the male and female providers (MNPs and FNPs) in terms of the frequency of requests for contraception encountered during their medical practice, which was “very frequent” or “frequent” for 57% of the women and “rare”, “very rare” or even “never” for 58.8% of the men (Table 5). Regarding the existing contraceptive arsenal, the FNPs felt more likely to be unable to answer patients’ questions than the MNPs (non-significant difference).

Among the new providers, 247 people (82.3% of the sample) claimed to be interested in participating in training courses regarding male contraception.

The advantages and disadvantages of TMC for new fathers and new providers are presented in Table 6.

**Table 6:** Thermal male contraception: Knowledge and hypothetical acceptability among the new providers as users and as providers–Comparison with the new fathers.

N° question	THERMAL MALE CONTRACEPTION	*New fathers*	MNPs	FNPs	NPs	MNPs	FNPs	NPs	p-value
				**FOR THEIR PATIENTS**	**= AS PROVIDERS**	**FOR THEM =**	**AS USERS**		
Q1.21 Q2M17 Q2F17	**ADVANTAGES OF TMC**	***n = 303 (%)***	**n = 97 (%)**	**n = 203 (%)**	**n = 300 (%)**	**n = 97 (%)**	**n = 203 (%)**	**n = 300 (%)**	
	Environmental	*87 (28*.*6)*	41 (42.3)	102 (50.2)	143 (47.7)	43(44.3)	106 (52.2)	149 (49.7)	
	Inexpensive	*61 (20*.*1)a*	59 (60.8)	124 (61.1)	183 (61)ad	48 (49.5)	99 (48.8)	147 (49)d	a,d : p<0.01
	No adverse effects	*117 (38*.*5)*	43 (44.3)	113 (55.7)	156 (52)	36 (37.1)	108 (53.2)	144 (48)	
	Efficiency	*60 (19*.*7)*	11 (11.3)	27 (13.3)	38 (12.7)	17 (17.5)	29 (14.3)	46 (15 .3)	
	Non-Hormonal	*110 (36*.*2)*	70 (72.2)	150 (73.9)	220 (73.3)	65 (67)	131 (64.5)	196 (65 .3)	
	Natural	*158 (52)*	54 (55.7)	145 (71.4)	200 (66.7)	52 (53.6)	124 (61.1)	176 (58.7)	
	Reversible	*109 (35*.*9)*	59 (60.8)	133 (65.5)	192 (64)	60 (61.9)	136 (67)	196 (65.3)	
	Other	*27 (8*.*9)*	1 (1)	3 (1.5)	4 (1.3)	0	1 (0.5)	1(0.3)	
	None	*NA*	6 (6.2)	3 (1.5)	9 (3)	6 (6,2)	6 (3)	12(4)	
Q1.22 Q2M18 Q2F18	**DISADVANTGES OF TMC**	***n = 303 (%)***	**n = 97 (%)**	**n = 203 (%)**	**n = 300 (%)**	**n = 97 (%)**	**n = 203 (%)**	**n = 300 (%)**	
	Delayed effectiveess	*94 (30*.*9)*	57 (59.4)	130 (64)	187 (62.3)	48 (49.5)	115 (6.7)	163 (54.3)	
	Delayed reversibility	*64 (21*.*1)*	37 (38.5)	100 (49.3)	137 (45.7)	39 (40.2)	88 (43.3)	127 (42 .3)	
	Time required for wear (15h/ day)	*170 (55*.*9)*	75 (78.1)	159 (78.3)	234 (78)	75 (77.3)	151 (74.4)	226 (75.3)	
	Aesthetic	*67 (22)*	44 (45.8)	119 (58.6)	163 (54.3)	35 (36.1)	93 (45.8)	128 (42.7)	
	Uncomfortable	*118 (38*.*8)*	52 (54.2)	91 (44.8)	143 (47.7)	64 (66)	88 (43.3)	152 (50.7)	
	Must be worn without fail	*131 (43*.*1)*	65 (67.7)	150 (69)	215 (71.7)	65 (67)	120 (59.1)	185 (61.7)	
	Loss of confidence	*103 (33*.*9)*	51 (53.1)	112 (55.2)	163 (54.3)	50 (51.5)	124 (61.1)	174 (58)	
	Damage to virility	*41 (13*.*5)a*	29 (30.2)	68 (33.5)	97 (32.3)a	17 (17.5)b	44 (21.7)b	61 (20.3)	a : p<0.01 b : NS
	STI risks	*51 (16*.*8)*	0	95 (46.8)	95 (31.7)	29 (29.9)	6 (3)	35 (11.7)	
	Other	*27 (8*.*9)*	5 (5.2)	0	5 (1.7)	2 (2.1)	6 (3)	8 (2.7)	
Q2M29 Q2F27	**WOULD YOU BE WILLING TO RECOMMEND TMC?**		**n = 97(%)**	**n = 203 (%)**	**n = 300 (%)**				
	YES	*NA*	58 (59.8)	127 (62.6)	185 (61.7)	NA	NA	NA	
Q1.23 Q2M19 Q2F19	**WOULD YOU BE WILLING TO TRY TMC?**	***n = 304 (%)***				**n = 97 (%)**	**n = 203 (%)**	**n = 300 (%)**	
	I would totally agree	*22 (7*.*2)ac*	NA	NA	NA	5 (5.2)bc	23 (11.3)bc	28 (9.3)a	a,c : p<0.01 b : p = 0.02
	I would rather agree	*67 (22)ac*	NA	NA	NA	25 (25.8)c	68 (33.5)c	93 (31)a	a : p = 0.012 c : p<0.01
	I would rather not agree	*92 (30*.*3)*	NA	NA	NA	38 (39.1)	83 (40.9)	121 (40.3)	
	Not at all	*123 (40*.*5)*	NA	NA	NA	29 (29.9)	29 (14.3)	58 (19.3)	

MNPs: Male new providers; FNPs: Female new providers; NPs: New providers; Column 1: The numbers of the questions in the questionnaire are presented in S1–S4 Files; Q1: questionnaire for new fathers, Q2M: questionnaire for MNPs; Q2F: questionnaire for FNPs; n = effective; HMC: Hormonal male contraception; TMC: Thermal male contraception; p < 0.05 significance threshold; a: Comparison between new fathers and new providers; b: Comparison between MNPs and FNPs; c: Comparison between new fathers + MNPs (men) and FNPs (women); d: Comparison between NPs as providers and NPs as users; NS: Not significant.

In general, for the new providers, regardless of sex and personal or prescriber experience, the main benefits of TMC were its non-hormonal, natural and reversible nature. The absence of side effects was the second greatest benefit according to the new fathers, while this was the fourth-mentioned consideration for the new providers.

When the new providers acted as prescribers, they would more often emphasize the economic considerations for their patients, although this factor was not as important for the providers when they acted as users.

The two main disadvantages according to the new providers were identical to those of the new fathers: namely, the duration of undergarment wear and the requirement for daily wear of the undergarment.

The new providers were significantly more concerned about damage to virility as prescribers (32.3%) than the new fathers as potential users (13.5%, p<0.01). When they considered themselves personally as potential users, only 20.3% of the male and female new providers emphasized this concern.

The new providers were more likely to recommend this method of contraception to their patients (as a prescriber) than to use it themselves, with 61.7% reporting that they were prepared to recommend TMC and only half reporting that they were likely to “strongly agree” or “generally agree” to the use of TMC in their personal lives (31% of MNPs and 44.3% of FNPs).

## Discussion

The main objective of our study was to evaluate knowledge regarding male contraception, particularly thermal male contraception (TMC), and its hypothetical acceptability in two populations: new fathers and new male and female providers.

### Knowledge and contraceptive practices

Most new fathers were aware of condoms and the withdrawal method, half of them had heard of vasectomy, and few were aware of newer male contraceptive methods (TMC/HMC = 14%). TMC was the least-known male contraceptive method in both populations in our study; however, it was better known by new providers than by new fathers (15% versus 2.6%, p < 0.01). Male providers appear to be better informed than female providers regarding “new” methods (HMC/TMC), particularly TMC (25.8% versus 9.9%, p < 0.01), suggesting that awareness is gained through personal experience rather than medical training.

Among the new providers, this lack of knowledge can be explained by a lack of initial training for physicians and lack of discussion regarding this subject in recent scientific literature. Many publications have reported “new” methods of male contraception and present hormonal methods versus non-hormonal methods [33–36]. Unfortunately, TMC is only rarely reported [7,14,37], and the only non-hormonal methods mentioned are potential “chemical” methods that remain under investigation.

Among the new fathers, this lack of knowledge can be explained by a lack of information from medical providers and a lack of representation in the general media.

The lack of knowledge in France regarding forms of male contraception and their mode of administration has already been identified [6,38] as a major obstacle to implementation and acceptability, and this study highlights the importance of information regarding this subject in both general and medical literature.

The most widely known male contraceptive method is the condom as previously reported [3], followed by vasectomy and withdrawal for new providers and withdrawal and then vasectomy for new fathers. Indeed, vasectomy was the least-known method among the new fathers, and it is not a common procedure in France because it has been legal only since July 4, 2001; fifty years of prohibition have strongly limited its development [39].

The couples in our study used less female contraception and more male contraception than the couples in a recent study performed in France regarding “contraceptive practices and the involvement of men in the contraceptive decision” [1].

Indeed, in our study, 62% of the new fathers reported using a female contraceptive method versus 43% reporting use of a male-oriented method, which included 30% of those using condoms.

Le Guen *et al*. [1] observed that in a sample of 1,776 men aged 15 to 49 in more or less stable relationships, 71.7% were using a female contraception method and 20.4% were using a male contraception method, including condom use in 18.9% of these men (12% of whom were living with their partners). Our population included stable couples with higher levels of education than Le Guen *et al*.’s population and the French general population [40]. As reported by Le Guen *et al*., men with a higher education level are more likely to use a male contraceptive method.

Therefore, condoms are used as a method of contraception among stable couples and not only as a method of protection against sexually transmitted infections (STIs) in casual relationships. In addition, the use of male contraception in the population is already widespread.

Finally, the response rate was significantly (p < 0.01) higher in the new fathers than that in the new providers, which can be explained by the recruitment method including hand-delivered questionnaires provided directly by the investigator that were later collected. Furthermore, a better response rate was observed for gynaecologists than for general practitioners, which may indicate higher contraceptive use among the gynaecologists.

### Interest in male contraception and willingness to share contraceptive responsibility

In our study, most participants (new fathers and new providers) expressed desire for more choices in terms of male contraception and more information. This desire was more pronounced in the new providers than that in the new fathers. Notably, 96% of the female providers asked for more information and 89.7% asked for more choices. This greater interest among women reflects the burden of contraceptive responsibility that they currently bear. Therefore, women want to share this burden with their partners. These results are consistent with data in the literature in France and in many countries, which show a positive attitude towards male contraception and shared contraceptive responsibility among both men and women and a true awareness of the importance of contraception [2].

In our study, only 15 of 203 female participants (approximately 7%) were reluctant to use male contraception because of a lack of confidence in the partner.

Finally, 74% of the female participants were in favour of using male contraception. These results are consistent with those of a study on the acceptability of HMC [41] in which only 36 of 1,895 women (2%) from 3 different regions (South Africa, China and Scotland) would not trust their partners. In the same study, 84% of these women agreed that more egalitarian sharing of contraceptive responsibility is warranted [41]. All these results suggest that women are willing to trust their partners with contraceptive responsibility.

Men are heavily involved in contraceptive decisions in their relationships. Indeed, among the new fathers, the choice of contraception was made by both female and male partners in 67% of the couples, accounting for two-thirds of the subjects and reflecting the clear involvement of men in stable relationships. Moreover, very few participants (13% and 10% of the new fathers and MNPs, respectively) thought that contraception was “a woman’s issue”, reinforcing previous findings. In the studies led by Martin *et al*. [2] and Heinemann *et al*. [3], contraceptive decision-making was shared in 54 to 80% of couples depending on the country of origin, with France holding the last position (54.1%) [2]. In our study, the two main incentives for male contraceptive use according to the men were to relieve their partner of the contraceptive responsibility and to avoid the side effects related to female contraception.

Our study corroborates the data in the literature and indicates an emerging “general willingness from many men around the world to take more responsibility for contraception” [2].

In our study, a statistical link was observed between willingness to use male contraception within a relationship and educational or social and professional levels, although an association was not observed with a negative experience related to contraception [42].

Similar findings have been shown for HMC in two studies by Heinemann *et al*. and Martin *et al*. [2,3]. We already noted that in the study by Le Guen *et al*. [1], men with higher levels of education were more likely to use a male contraceptive method. In our study, the new providers (100% with at least a master’s degree) had higher educational levels than the new fathers (35% with at least a master’s degree), and our two populations overall had higher education levels than those of Heinemann's study [3].

Among the male and female providers, a positive attitude towards male contraception was also related to their contraceptive experiences because when one of the partners in a couple had suffered side effects from contraception, this influenced their opinions. The men and women in these populations who were willing to use a male contraceptive method in their relationships were those who had already experienced side effects at home or with their partners. Similar findings have been reported within the French population by Le Guen *et al*. [1], who reported “having a pregnancy that was terminated influenced men's contraceptive practices”.

In our study, religion seemed to have a minor effect on willingness to use male contraception. Within MNPs, more religious men indicated that they were not interested in managing the couple's contraceptive practice via male contraception. This result can be compared with those of another French study [43] regarding the complex relationship between religiousness and sexual behaviours. The study concluded that “sexually experienced adolescents who reported regular religious practice were less likely to use contraception” [43].

### Attitude towards TMC

Approximately one-third of each population (NFs, MNPs and FNPs) would “totally agree” or “generally agree” to try TMC, with 30% of the new fathers and 31% of the male new providers agreeing versus 44% of the female new providers. Moreover, the men who would “totally agree” or “generally agree” to try TMC had more often experienced side effects of contraception than the men who would “rather not” agree or “not agree at all”; among the new fathers, those who would agree to try TMC would also more often accept a vasectomy. These results highlight the influences of sex and experience with contraception and exclude the influences of educational level and medical training on the willingness to use TMC.

Furthermore, women may be more accepting of male contraception because they are not affected by the practical constraints imposed by TMC. In addition, women are directly affected by the contraceptive responsibility that they wish to share. These results are consistent with those of a study published in 2009 [4] concerning attitudes towards a male contraceptive pill (HMC) in which a significantly more positive “attitude” was observed among women than men.

The main reported advantageous features of TMC were as follows: non-hormonal, natural, reversible and without side effects. The efficiency of TMC was ranked last by the 3 populations. The participants probably accepted this method as efficient (perhaps because it was introduced to them by physicians) and then considered that the “benefits” are the features that differentiate TMC from the other methods.

Our results emphasize the importance of a non-hormonal contraceptive method for new fathers and future providers. A study on the psychosocial factors related to male medical contraception [38] showed that the lack of biological and medical knowledge regarding male contraception led to transposition of male to female contraception, such as the idea of a male hormonal pill [2].

The growing interest in non-hormonal and more “natural” methods may be partly explained by the controversy over the third- and fourth-generation pills that were distributed in France in 2012–2013, which resulted from a complaint against a pharmaceutical company filed by a young patient who suffered a stroke while using a third-generation pill prescribed in a context of a family history of venous thrombosis [44]. The controversy resulted in reluctance to use birth control pills and changes in the contraceptive landscape [45]. However, these changes in attitude are also a reflection of the side effects associated with female hormonal contraception.

The difficulty of accepting TMC appeared to be predominantly based on practical constraints, including daily and prolonged wearing of the undergarment and the need to remember to wear the undergarment. Comfort was only the 3^rd^ highest concern.

These disadvantages arise from an imagined expectation. Data regarding TMC comfort are limited because the participants in various TMC trials “were all voluntary demanders of male contraception (thermal and not-hormonal), which may explain the low rate of reported annoyance” [14,46].

### Prescribers’ perspectives

To assess the acceptability of TMC, we asked the new providers for their perspectives as prescribers.

Knowledge of TMC was similar between the men and women, and most of their responses showed no differences, with sex appearing to have little or no influence on the physicians’ attitudes towards contraception.

Women were more often confronted with contraceptive demands and more often felt deprived than did men because of the current therapeutic arsenal. In our sample, more women were gynaecologists; therefore, the female participants may have had greater exposure to contraceptive topics.

Male contraception is rarely offered. Apart from condoms, 52% of the practitioners never offered any other contraceptive methods because they did not think of other methods. When they did, they frequently proposed vasectomy, but they did not offer TMC or HMC because of a lack of knowledge and training on this topic rather than a lack of interest.

A significant gap is apparent in the initial training of contraceptive specialists (gynaecologists) and primary care physicians represented by general practitioners. These results allow us to highlight one of the important general constraints of the dissemination of information regarding male contraception [6], especially for TMC, which is the lack of knowledge and therefore the lack of propagation by prescribers.

Finally, several of the projections of the acceptability of TMC among patients (new fathers) by the physicians (new providers) were false.

Indeed, the physicians felt that the main benefits of TMC for their patients were in the order non-hormonal > natural > reversible > economical, whereas the new fathers ranked the lack of side effects in the second position and the economic factor in the penultimate position. Regarding the economical aspect, these responses may be influenced by the fact that the new fathers group had a very low unemployment rate and included a high proportion of men in a high socio-professional category (Q1.3, S2 File).

For the disadvantages, differences were observed, and according to the new fathers, discomfort appeared in the 3^rd^ position whereas physicians ranked it last for their patients. Delay in action was not as important to the new fathers as it was for the providers.

In addition, the providers were more likely to fear damage to virility for their patients than the new fathers, with approximately one-third of the prescribers expressing concern regarding this disadvantage of the TMC method compared to 13.5% of the new fathers. We expected greater reticence in connection with virility. In fact, in the collective imagination, “medical contraception is a form of damage to virility”, which is an idea that has also been shared by women [38].

Moreover, 61.7% of the physicians were prepared to recommend TMC as a male contraceptive method to their patients. These results are encouraging for male contraception and show that prescribers trust this method after receiving proper information.

In 2016, a medical thesis was conducted in France among 310 prescribing physicians (general practitioners, family planning physicians, gynaecologists and andrologists) to determine the concerns and motivations for the expansion of HMC, and this study concluded that 87.7% of physicians were in favour of commercializing HMC. An oral form alone was the most accepted, although one-third of the doctors said that they were prepared to prescribe it in any form [47].

The doctors were very supportive of HMC but felt that they were not appropriately informed, and 97% wanted to receive more information (92% of the new providers in our study) [47]. These results are consistent with the other results of our study.

### Strengths and limitations

This multicentre study was performed in 7 different public and private facilities in the Bouches du Rhone Department and with a consistent sample (305 new fathers and 300 doctors and residents). The questionnaires were anonymous, which allowed the participants greater freedom of response. Many studies have examined the acceptability of HMC [2,3,11,29,30,41,42], but ours uniquely addressed TMC. Finally, our study permitted evaluation of TMC from different perspectives: i) Potential users and prescribers; and ii) women (physicians only) and men.

The present study constitutes the first response to the limited data available on the opinions of providers and women in the international literature [41].

The limitations of this study included a difference in the recruitment methods (distribution of paper forms versus forms sent by email) between the two populations, which could lead to recruitment bias (bias of selection). Similarly, the new fathers were in a favourable position for contraceptive use but also far from an unwanted pregnancy situation.

## Conclusions

We performed the first study to evaluate the acceptability of TMC among potential users and potential prescribers to assess their concerns and motivations regarding TMC. Our results indicate that among those who participated in our study, 30 to 45% would be interested in TMC (30% of the new fathers and 45% of the female new providers), and between 58% (new fathers) and 74% (female new providers) would be interested in male contraception in general. The men wanted to relieve women of their contraceptive responsibility and the potential deleterious effects of female contraceptives [11].

TMC presents many advantages for potential users and prescribers and fulfils many of the criteria sought in a contraceptive method, i.e., non-hormonal, effective, reversible, without side effects and economical. For several of the respondents in stable relationships, TMC may represent a possible contraceptive method after childbirth [6] according to their “intention of use”. The main obstacles identified are the lack of knowledge among potential users, reservations about the conditions of use and lack of training of physicians regarding male contraception and TMC.

## Supporting information

S1 File Information page about thermal male contraception. This information page was included in each questionnaire.
(DOCX)

S2 File Questionnaire for new fathers. Number of the question, question, answer, number of missing answers.
(DOCX)

S3 File Questionnaire for male new providers. Number of the question, question, answer, number of missing answers.
(DOCX)

S4 File Questionnaire for female new providers. Number of the question, question, answer, number of missing answers.
(DOCX)
