# Supplementary material for: Are men ready to use thermal male contraception? Acceptability in two French populations: New fathers and new providers
Source: PLoS One. 2018 May 29;13(5):e0195824. doi: 10.1371/journal.pone.0195824 (PMC5973589; doi:10.1371/journal.pone.0195824)
Supplement: S1 File — This information page was included in each questionnaire. (DOCX) [file pone.0195824.s001.docx]

**THERMAL MALE CONTRACEPTION**

**All studies on contraception are converging and show that women and men are ready to change their habits and to give way to male contraception in order to share responsibility for fertility.
Apart from condom and withdrawal, new methods of contraception for men have emerged: hormonal contraception (weekly intramuscular injection of testosterone) and thermal contraception.**

**What is thermal contraception?**

The testes are normally at a temperature from 2 to 4°C below the body temperature, to ensure the production of spermatozoa (Fig. 1). The most widely tested method of thermal contraception is to raise the testicular temperature of about 2°C.

**How the temperature of testicle raised?**

By using an undergarment with an orifice at the root of the penis which allows the testicles to be brought into supra-scrotal (Fig. 2) where their temperature is increased by 2 ° C.

*
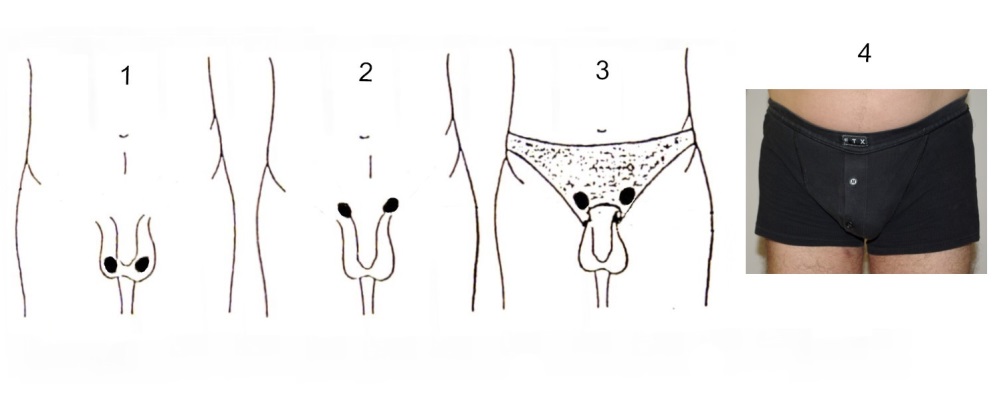
*

In practice, the man puts the specific undergarment like a classic undergarment. He then passes his penis through the orifice, then the skin of the bursa, which leads positioning the testicles in the desired location (Fig. 3). The underwear is of no discomfort, creates no friction; It is possible to wear a boxer over it (Fig. 4).
When worn for 15 hours a day, daily sperm production decreases to a contraceptive level in 2 to 4 months, it remains at this level only if the daily wearing is continued.
 **Is it effective and reversible?**

Studies were conducted in 37 couples who used the thermal method as the only contraception of the couple for 6 to 24 months. They showed a very good efficiency (no pregnancy), a return to normal sperm production in 3 to 6 months and fertility (reversibility), and the absence of side effects (safety).

[Shafik A. Testicular suspension as a method of male contraception: technique and results. Adv Contr Deliv Syst. 1991; VII: 269-279. Mieusset R, Bujan L. The potential of mild testicular heating as a safe, effective and reversible contraceptive method for men. Int J Androl. 1994 ;17: 1861-91.]

**Would you be likely to use this method of male contraception in your couple?**
