# Supplementary material for: Are men ready to use thermal male contraception? Acceptability in two French populations: New fathers and new providers
Source: PLoS One. 2018 May 29;13(5):e0195824. doi: 10.1371/journal.pone.0195824 (PMC5973589; doi:10.1371/journal.pone.0195824)
Supplement: S2 File — Number of the question, question, answer, number of missing answers. (DOCX) [file pone.0195824.s002.docx]

*S2 File Questionnaire new fathers*

**1.1 You**

| Q1.1 | **How old are you?** | 34,07 ±6,02 yo | 1 |
| --- | --- | --- | --- |

| Q1.2 | **What is your educationnal background?** |  |  |
| --- | --- | --- | --- |

|  | Before BAC | 63 (20,7%) |  |
| --- | --- | --- | --- |
|  | BAC | 46 (15,1%) |  |
|  | BAC +1 à BAC + 4 | 93 (31,5%) |  |
|  | BAC + 5 or more | 100 (32,8%) |  |

| Q1.3 | **What is your job ?** | **SocioProfessional Category ISCO08**   \| 0 \| 0.7% \| \| --- \| --- \| \| 1 \| 14.1% \| \| 2 \| 31.1% \| \| 3 \| 17.4% \| \| 4 \| 2.6 % \| \| 5 \| 14.1% \| \| 6 \| 0.7% \| \| 7 \| 9.8% \| \| 8 \| 3.3% \| \| 9 \| 3.0% \| |  |
| --- | --- | --- | --- | --- | --- | --- | --- | --- | --- | --- | --- | --- | --- | --- | --- | --- | --- | --- | --- | --- | --- | --- | --- |

| Q1.4 | **Do you practice a religion?** |  |  |
| --- | --- | --- | --- |
|  | Yes | 106 (34,8%) |  |
|  | No | 199 (65,2%) |  |

| Q1.5 | **Are you in a relationship?** |  |  |
| --- | --- | --- | --- |
|  | Yes | 304 (99,7%) |  |
|  | No | 1 (0,3%) |  |

| Q1.6 | **If you answered yes, for how long?** | 7,65 ± 4,32 yo | 1 |
| --- | --- | --- | --- |

| Q1.7 | **How many children have you got?** | 1,65 ± 0,93 |  |
| --- | --- | --- | --- |

| Q1.8 | 8. **Would you like to have other children (or a first)?** |  | 2 |
| --- | --- | --- | --- |
|  | Yes | 198 (65,3%) |  |
|  | No | 105 (34,7%) |  |

**1.2.Contraception and you**

| Q1.9 | **Have you ever had side effects due to contraception?** |  | 4 |
| --- | --- | --- | --- |
|  | Yes i have | 4 (1,3%) |  |
|  | Yes my partner has | 65 (21,6%) |  |
|  | No for none of us | 232 (77,1%) |  |

| Q1.10 | **Have you ever had a previous unwanted pregnancy while on birth control?** |  | 4 |
| --- | --- | --- | --- |
|  | Yes | 38 (12,6%) |  |
|  | No | 263 (87,4%) |  |

**Before current pregnancy**

| Q1.11 | **Which type of contraception did you use?** |  |  |
| --- | --- | --- | --- |
|  | Pill ( oral contraception) | 154 (51,5%) | 6 |
|  | IUD ( intrauterine device) | 29 (9,7%) | 6 |
|  | Nexplanon | 6 (2%) | 6 |
|  | Rhythm Method of Family Planning | 0 | 6 |
|  | Female condom | 1 (0,3%) | 6 |
|  | Cervical cap | 0,00% | 6 |
|  | Male condom | 91 (30,4%) | 6 |
|  | Withdrawal | 40 (13,4%) | 6 |
|  | Hormonal male contraception | 0,00% | 6 |
|  | Male contraception by hyperthermia | 0,00% | 6 |
|  | None | 51 (17,1%) | 6 |
|  | Other | 17 (5,7%) | 6 |

| Q1.12 | **Who made that choice?** |  |  |
| --- | --- | --- | --- |
|  | You | 10 (3,3%) |  |
|  | Your partner | 87 (28,5%) |  |
|  | Both | 203 (67,7%) |  |

**After the birth of your child when necessary:**

| Q1.13 | **Would you use a contraception?** |  | 8 |
| --- | --- | --- | --- |
|  | Yes | 241 (81,1%) |  |
|  | No | 56 (18,9%) |  |

| Q1.14 | **If you answered yes, which one(s)?** |  | 47 |
| --- | --- | --- | --- |
|  | Pill ( oral contraception) | 111 (43%) | 47 |
|  | IUD ( intrauterine device) | 75 (29,1%) | 47 |
|  | Nexplanon | 11 (4,3%) | 47 |
|  | Rythm Method of Family Planning | 1 (0,4%) | 47 |
|  | Female condom | 3 (1,2%) | 47 |
|  | Cervical cap | 0,0% | 47 |
|  | Male condom | 63 (24,4%) | 47 |
|  | Withdrawal | 15 (5,8%) | 47 |
|  | Male Hormonal Contraception | 1 (0,4%) | 47 |
|  | Male contraception by hyperthermia | 1 (0,4%) | 47 |
|  | None | 12 (4,7%) | 47 |
|  | Other | 24 (7,9%) | 47 |

**1.3. Male contraception**

| Q1.15 | **In the list below, what type of male contraception do you know?** |  |  |
| --- | --- | --- | --- |
|  | Condom | 298 (97,7%) |  |
|  | Withdrawal | 193 (63,3%) |  |
|  | Vasectomy | 146 (47,9%) |  |
|  | Hormonal male contraception | 34 (11,3%) |  |
|  | Male contraception by hyperthermia | 8 (2,6%) |  |
|  | None | 7 (2,3%) |  |
|  | Others (please specify) | 4 (1,3%) |  |

| Q1.16 | **Would you agree to use a male contraception?** |  |  |
| --- | --- | --- | --- |
|  | Oui | 178 (58,4%) |  |
|  | Non | 127 (41,6%) |  |

| Q1.17 | **If you answered YES (question 16), what is your main reason ? (One answer possible)** | n=178 | 125 |
| --- | --- | --- | --- |
|  | To share contraceptive responsability | 91 (51,4%) | 125 |
|  | To have an extra safety to avoid pregnancy | 27 (15,3%) | 125 |
|  | To avoid having a child aknowlingly | 14 (7,9%) | 125 |
|  | To avoid side effects due to female contraception | 67 (37,9%) | 125 |
|  | Not to take the risk of having a child with another partner | 4 (2,3%) | 125 |
|  | Other ( please specify ) | 1 (0,6%) | 125 |

| Q1.18 | **If you answered No (question 16), what is your main reason? ( One answer possible)** | N= 127 | 176 |
| --- | --- | --- | --- |
|  | Unconvenient | 27 (21,6%) | 176 |
|  | Because of side effects | 18 (14,4%) | 176 |
|  | Contraception belongs to women | 16 (12,7%) | 176 |
|  | It Damages my virility | 9 (7,2%) | 176 |
|  | Not interested at all | 49 (39,2%) | 176 |
|  | Other (precise)…… | 23 (18,4%) | 176 |

| Q1.19 | **Have you ever heard of male contraception by hyperthermia?** |  |  |
| --- | --- | --- | --- |
|  |  | 17 (5,6%) |  |
|  |  | 288 (94,4%) |  |

Please read this short following information about the male contraception by hyperthermia and answer to question 20 to question 27.

**TMC INFORMATION (Annex 2)**

| Q1.20 | **Which type of male contraception would you be willing to use? (Only one answer)** | | 1 |
| --- | --- | --- | --- |
|  | Condom | 196 (64,5%) |  |
|  | Withdrawal | 49 (16,1%) |  |
|  | Vasectomy | 8 (2,6%) |  |
|  | Hormonal Male contraception | 5 (1,6%) |  |
|  | Male contraception by hyperthermia | 30 (9,9%) |  |
|  | None | 18 (5,9%) |  |
|  | Other (precise) | 6 (2,0%) |  |

| Q1.21 | **As to male contraception by hyperthermia, what would the pros (advantages) be? ( several answer possible)** | |  |
| --- | --- | --- | --- |
|  | Environmental | 87 (28,6%) | 1 |
|  | Inexpensive | 61 (20,1%) | 1 |
|  | No adverse effect | 117 (38,5%) | 1 |
|  | Efficient | 60 (19,7%) | 1 |
|  | Non-hormonal | 110 (36,2%) | 1 |
|  | Natural method | 158 (52%) | 1 |
|  | Reversible | 109 (35,9%) | 1 |
|  | Other (please specify) | 27 (8,9%) | 1 |

| Q1.22 | **As to male contraception by hyperthermia, what would the cons (disadvantages) be? ( several answers possible)** |  |  |
| --- | --- | --- | --- |
|  | Delayed effectiveness | 94 (30,9%) | 1 |
|  | Delayed reversibility | 64 (21,1%) | 1 |
|  | Time required for wear ( 15h per day ) | 170 (55,9%) | 1 |
|  | Aesthetic apperance (embarassment) | 67 (22%) | 1 |
|  | Uncomfortable | 118 (38,8%) | 1 |
|  | Must be wom without fail | 131 (43,1%) | 1 |
|  | Loss of confidence | 103 (33,9%) | 1 |
|  | Makes felle less virile | 41 (13,5%) | 1 |
|  | STIs risks | 51 (16,8%) | 1 |
|  | Others (please specifify) | 27 (8,9%) | 1 |

| Q1.23 | **Would you accept to try that type of male contraception?** |  |  |
| --- | --- | --- | --- |
|  | I would totally accept | 22 (7,2%) |  |
|  | I would generally accept | 67 (22%) |  |
|  | I would generally not accept | 92 (30,3%) |  |
|  | Not at All | 123 (40,5%) |  |

| Q1.24 | **Which period of your man life would be the most appropriate to use a male contraception by hyperthermia? ( one answer only )** | N=89 (only interested sample) | 4 |
| --- | --- | --- | --- |
|  | Single | 12 (4%) | 4 |
|  | Unstable relationship | 8 (2,7%) | 4 |
|  | Before having the first child | 17 (5,6%) | 4 |
|  | Between two children | 40 (13,3%) | 4 |
|  | After children's birth | 64 (21,3%) | 4 |
|  | If female contraception is impossible in your relationship | 59 (19,6%) | 4 |
|  | No opinion | 99 (32,9%) | 4 |
|  | Other | 16 (5,3%) | 4 |

| Q1.25 | **Would you like to have more information about male contraception in general?** |  | 4 |
| --- | --- | --- | --- |
|  | Yes | 130 (43,2%) |  |
|  | No | 171 (56,8%) |  |

| Q1.26 | **Would you like a larger variety of choices in male contraception?** |  | 4 |
| --- | --- | --- | --- |
|  | Yes | 163 (54,2%) |  |
|  | No | 138 (45,8%) |  |

| Q1.27 | **Could you be interested to have a vasectomy?** |  | 4 |
| --- | --- | --- | --- |
|  | Yes | 46 (15,3%) |  |
|  | No | 255 (84,7%) |  |

**Thanks for your involvement!**
