# Supplementary material for: Are men ready to use thermal male contraception? Acceptability in two French populations: New fathers and new providers
Source: PLoS One. 2018 May 29;13(5):e0195824. doi: 10.1371/journal.pone.0195824 (PMC5973589; doi:10.1371/journal.pone.0195824)
Supplement: S3 File — Number of the question, question, answer, number of missing answers. (DOCX) [file pone.0195824.s003.docx]

*S3 File Questionnaire for Male New Providers*

**1.1 You, as a user of a contraception**

| Q2M1 | **How old are you?** | 27,65 ± 2,41 | 1 |
| --- | --- | --- | --- |

| Q2M2 | **What is your medical specialty?** |  |  |
| --- | --- | --- | --- |
|  | Medical Gynaecology | 0,0% |  |
|  | Gyneacolgy-obstetrics | 3 (3,1%) |  |
|  | General medical practice | 94 (96,9%) |  |

| Q2M3 | **Where do you practice?** |  |  |
| --- | --- | --- | --- |
|  | Private practice | 29 (29,9%) |  |
|  | Health centre | 4 (4,1%) |  |
|  | Hospital | 64 (66%) |  |

| Q2M4 | **Do you practice a religion?** |  |  |
| --- | --- | --- | --- |
|  | Yes | 19 (19,6%) |  |
|  | No | 78 (80,4%) |  |

󠄀

| Q2M5 | **Are you in a relationship?** |  |  |
| --- | --- | --- | --- |
|  | Yes | 74 (76,3%) |  |
|  | No | 23 (23,7%) |  |

| Q2M6 | **If you answered yes, for how long?** | 3,84 ± 3,16 | 22 |
| --- | --- | --- | --- |

| Q2M7 | **How many children have you got?** | 0,07 ± 0,30 |  |
| --- | --- | --- | --- |

| Q2M8 | **Would you like to have other children (or a first)?** |  |  |
| --- | --- | --- | --- |
|  | Yes | 82 (84,5%) |  |
|  | No | 15 (15,5%) |  |

**1.2 You and contraception**

| Q2M9 | **Have you ever had side effects due to contraception?** |  |  |
| --- | --- | --- | --- |
|  | Yes i have | 2 (2,1%) |  |
|  | Yes my Partner has | 50 (51,5%) |  |
|  | No for none of us | 45 (46,4%) |  |

| Q2M10 | **Have you ever had a previous unwanted pregnancy while on birth control?** |  |  |
| --- | --- | --- | --- |
|  | Yes | 13 (13,4%) |  |
|  | No | 84 (86,6%) |  |

**1.3 Male contraception**

| Q2M11 | **In the list below, what type of male contraception do you know?** |  |  |
| --- | --- | --- | --- |
|  | Condom | 96 (99%) |  |
|  | Withdrawal | 63 (64,9%) |  |
|  | Vasectomy | 83 (85,6%) |  |
|  | Hormonal male contraception | 31 (32%) |  |
|  | Male contraception by hyperthermia | 25 (25,8%) |  |
|  | None | 0%%) |  |
|  | Others (please specify) | 4 (4,1%) |  |

| Q2M12 | **Would you agree to use a male contraception?** |  |  |
| --- | --- | --- | --- |
|  | Oui | 68 (70,1%) |  |
|  | Non | 29 (29,9%) |  |

| Q2M13 | **If you answered YES (question 16), what is your main reason? (One answer possible)** | N=68 |  |
| --- | --- | --- | --- |
|  | To share contraceptive responsability | 41 (60,3%) | 26 |
|  | To have an extra safety to avoid pregnancy | 3 (4,4%) | 26 |
|  | To avoid having a child aknowlingly | 7 (10,3%) | 26 |
|  | To avoid side effects due to female contraception | 16 (23,5%) | 26 |
|  | Not to take the risk of having a child with another partner | 0,0% | 26 |
|  | Other (please specify) | 1 (1,5%) | 26 |

| Q2M14 | **If you answered No (question 16), what is your main reason? ( One answer possible)** | N=29 |  |
| --- | --- | --- | --- |
|  | Unconvenient | 9 (31%) | 66 |
|  | Because of side effects | 9 (31%) | 66 |
|  | Contraception belongs to women | 3 (10,3%) | 66 |
|  | It Damages my virility | 2 (6,9%) | 66 |
|  | Not interested at all | 3 (10,3%) | 66 |
|  | Other (precise) | 3 (10,3%) | 66 |

| Q2M15 | **Have you ever heard of thermal male contraception?** |  |  |
| --- | --- | --- | --- |
|  | Yes | 28 (28,8%) |  |
|  | No | 69 (71,1%) |  |

Please read this short following information about the male contraception by hyperthermia and answer to question 16 to question 33.

**TMC INFORMATION Annex 2**

| Q2M16 | **Which type of male contraception would you be willing to use? (Only one answer)** | |  |
| --- | --- | --- | --- |
|  | Condom | 71 (73,2%) |  |
|  | Withdrawal | 3 (3,1%) |  |
|  | Vasectomy | 3 (3,1%) |  |
|  | Hormonal Male contraception | 2 (2,1%) |  |
|  | Male contraception by hyperthermia | 12 (12,4%) |  |
|  | None | 5 (5,2%) |  |
|  | Other (precise) | 1 (1%) |  |

| Q2M17 | **As to male contraception by hyperthermia, what would the pros (advantages) be? ( several answer possible)** | |  |
| --- | --- | --- | --- |
|  | Environmental | 43 (44,3%) |  |
|  | Inexpensive | 48 (49,5%) |  |
|  | No adverse effect | 36 (37,1%) |  |
|  | Efficient | 17 (17,5%) |  |
|  | Non-hormonal | 65 (67%) |  |
|  | Natural method | 52 (53,6%) |  |
|  | Reversible | 60 (61,9%) |  |
|  | Other (please specify) | 0,0% |  |

| Q2M18 | **As to male contraception by hyperthermia, what would the cons (disadvantages) be? ( several answers possible)** |  |  |
| --- | --- | --- | --- |
|  | Delayed effectiveness | 48 (49,5%) |  |
|  | Delayed reversibility | 39 (40,2%) |  |
|  | Time required for wear ( 15h per day ) | 75 (77,3%) |  |
|  | Aesthetic apperance (embarassment) | 35 (36,1%) |  |
|  | Uncomfortable | 64 (66%) |  |
|  | Must be wom without fail | 65 (67%) |  |
|  | Loss of confidence | 50 (51,5%) |  |
|  | Makes felle less virile | 17 (17,5%) |  |
|  | STIs risks | 29 (29,9%) |  |
|  | Others (please specifify) | 2 (2,1%) |  |

| Q2M19 | **Would you accept to try that type of male contraception?** |  |  |
| --- | --- | --- | --- |
|  | I would totally accept | 5 (5,2%) |  |
|  | I would generally accept | 25 (25,8%) |  |
|  | I would generally not accept | 38 (39,1%) |  |
|  | Not at All | 29 (29,9%) |  |

| Q1.20 | **Which period of your man life would be the most appropriate to use a male contraception by hyperthermia? (one answer only)** | N= 30 | 4 |
| --- | --- | --- | --- |
|  | Single | 1 (3,3%) |  |
|  | Unstable relationship | 3 (0,1%) |  |
|  | Before having the first child | 13 (43,3%) |  |
|  | Between two children | 9 (30%) |  |
|  | After children's birth | 0,0% |  |
|  | If female contraception is impossible in your relationship | 1 (3,3%) |  |
|  | No opinion | 2 (6,7%) |  |
|  | Other | 1 (3,3%) |  |

| Q2M21 | **Would you like a larger variety of choices in male contraception?** |  |  |
| --- | --- | --- | --- |
|  | Yes | 72 (74,2%) |  |
|  | No | 25 (25,8%) |  |

| Q2M22 | **Could you be interested to have a vasectomy?** |  |  |
| --- | --- | --- | --- |
|  | Yes | 30 (30,9%) |  |
|  | No | 67 (69,1%) |  |

- 1. **You, as a prescriber of contraception**

| Q2M23 | **Are you often asked to prescribe contraception?** |  |  |
| --- | --- | --- | --- |
|  | Very often | 5 (5,2%) |  |
|  | Often | 35 (36,1%) |  |
|  | Rarely | 29 (29,9%) |  |
|  | Very rarely | 14 (14,4%) |  |
|  | Never | 14 (14,4%) |  |

| Q2M24 | **Do you ever feel powerless as regards current means of contraception?** |  |  |
| --- | --- | --- | --- |
|  | Yes | 43 (44,3%) |  |
|  | No | 54 (55,7%) |  |

| Q2M25 | **How often do you propose male contraception?** |  |  |
| --- | --- | --- | --- |
|  | Very often | 1 (1%) |  |
|  | Often | 15 (15,5%) |  |
|  | Rarely | 21 (21,6%) |  |
|  | Very rarely | 23 (23,7%) |  |
|  | Never | 37 (38,1%) |  |

| Q2M26 | **Have you ever proposed other types of male contraception than condoms?** |  |  |
| --- | --- | --- | --- |
|  | Very often | 1 (1%) |  |
|  | Often | 5 (5,2%) |  |
|  | Rarely | 19 (19 ,6%) |  |
|  | Very rarely | 21 (21,6%) |  |
|  | Never | 51 (52,6%) |  |

| Q2M27 | **If you answered yes, which one(s)?** |  |  |
| --- | --- | --- | --- |
|  | Vasectomy | 37 (38,5%) | 1 |
|  | Hormonal male contraception | 3 (3,1%) | 1 |
|  | Thermal male contraception | 5 (5,2%) | 1 |
|  | None | 55 (57,3%) |  |
|  | Other (please specify) | 2 (2,1%) |  |

| Q2M28a | **If you do not propose vasectomy, why? (Several answers possible )** | N = 59 |  |
| --- | --- | --- | --- |
|  | I don’t know about it | 6 (11,1%) | 33 |
|  | I have no confidence in it | 1 (1,9%) | 33 |
|  | I don’t have any qualification to prescribe | 15 (7,8%) | 33 |
|  | I don’t know any appropriate colleague I could liaise with | 13 (24,1%) | 33 |
|  | I’m concerned about side effects | 6 (11,1%) | 33 |
|  | It never occurs to me | 23 (42,6%) | 33 |
|  | Other (please specify) | 9 (16,7%) | 33 |

| Q2M28 b | **If you do not propose hormonal male contraception, why? (Several answers possible)** | N=91 |  |
| --- | --- | --- | --- |
|  | I don’t know about it | 46 (52,9%) |  |
|  | I have no confidence in it | 9 (10,3%) |  |
|  | I don’t have any qualification to prescribe it | 27 (31%) |  |
|  | I don’t know any appropriate colleague I could liaise with | 14 (16,1%) |  |
|  | I’m concerned about side effects | 20 (23%) |  |
|  | It never occurs to me | 9 (10,3%) |  |
|  | Other (please specify) | 0,0% |  |

| Q2M28c | **If you do not propose thermal male contraception, why? (several answers possible)** | N= 93 |  |
| --- | --- | --- | --- |
|  | I don’t know about it | 58 (64,4%) | 5 |
|  | I have no confidence in it | 19 (21,1%) | 5 |
|  | I don’t have any qualification to prescribe it | 12 (13,3%) | 5 |
|  | I don’t know any appropriate colleague I could liaise with | 8 (8,9%) | 5 |
|  | I’m concerned about side effects | 7 (7,8%) | 5 |
|  | It never occurs to me | 17 (18,9%) | 5 |
|  | Other (please specify) | 2 (2,2%) | 5 |

| Q2M29 | **Would you be willing to recommend male contraception by hyperthermia?** |  |  |
| --- | --- | --- | --- |
|  | Yes | 58 (59,8%) |  |
|  | No | 39 (40,2%) |  |

| Q2M30 | **As to male contraception by hyperthermia, what would the pros (advantages) be FOR YOUR PATIENTS? ( several answer possible)** | |  |
| --- | --- | --- | --- |
|  | Environmental | 41 (42,3%) |  |
|  | Inexpensive | 59 (60,8%) |  |
|  | No adverse effect | 43 (44,3%) |  |
|  | Efficient | 11 (11,3%) |  |
|  | Non-hormonal | 70 (72,2%) |  |
|  | Natural method | 54 (55,7%) |  |
|  | Reversible | 59 (60,8%) |  |
|  | None | 6 (6,2%) |  |
|  | Other (please specify) | 1 (1%) |  |

| Q2M31 | **As to male contraception by hyperthermia, what would the cons (disadvantages) be FOR YOUR PATIENTS? ( several answers possible)** | |  |
| --- | --- | --- | --- |
|  | Delayed effectiveness | 57 (59,4%) | 1 |
|  | Delayed reversibility | 37 (38,5%) | 1 |
|  | Time required for wear ( 15h per day ) | 75 (78,1%) | 1 |
|  | Aesthetic apperance (embarassment) | 44 (45,8%) | 1 |
|  | Uncomfortable | 52 (54,2%) | 1 |
|  | Must be wom without fail | 65 (67,7%) | 1 |
|  | Loss of confidence | 51 (53,1%) | 1 |
|  | Makes felle less virile | 29 (30,2%) | 1 |
|  | STIs risks | 0,0% | 1 |
|  | Others (please specify) | 5 (5,2%) | 1 |

| Q2M32 | **Would you like to have more information about male contraception in general?** |  |  |
| --- | --- | --- | --- |
|  | Yes | 81 (83,5%) |  |
|  | No | 16 (16,5%) |  |

| Q2M33 | **Would you be interested to participate to a training course about male contraception?** |  |  |
| --- | --- | --- | --- |
|  | Yes | 74 (76,3%) |  |
|  | No | 23 (23,7%) |  |

**Thank you for your collaboration!**
