# Supplementary material for: Are men ready to use thermal male contraception? Acceptability in two French populations: New fathers and new providers
Source: PLoS One. 2018 May 29;13(5):e0195824. doi: 10.1371/journal.pone.0195824 (PMC5973589; doi:10.1371/journal.pone.0195824)
Supplement: S4 File — Number of the question, question, answer, number of missing answers. (DOCX) [file pone.0195824.s004.docx]

*S4 File Questionnaire for Female New Providers*

**1.1 You, as a user of a contraception**

| Q2F1 | **How old are you?** | 27,17 ±2,45 yo |  |
| --- | --- | --- | --- |

| Q2F2 | **What is your medical specialty?** |  |  |
| --- | --- | --- | --- |
|  | Medical Gynaecology | 5 (2,5%) |  |
|  | Gyneacolgy-obstetrics | 25 (12,3%) |  |
|  | General medical practice | 173 (85,2%) |  |

| Q2F3 | **Where do you practice?** |  |  |
| --- | --- | --- | --- |
|  | Private practice | 66 (32,5%) |  |
|  | Health centre | 11 (5,4%) |  |
|  | Hospital | 126 (62,1%) |  |

| Q2F4 | **Do you practice a religion?** |  |  |
| --- | --- | --- | --- |
|  | Yes | 57 (28,1%) |  |
|  | No | 146 (71,9%) |  |

| Q2F5 | **Are you in a relationship?** |  |  |
| --- | --- | --- | --- |
|  | Yes | 151 (74,4%) |  |
|  | No | 52 (25,6%) |  |

| Q2F6 | **If you answered yes, for how long?** | 4,13 ± 3,20 | 53 |
| --- | --- | --- | --- |

| Q2F7 | **How many children have you got?** | 0,13 ± 0,41 |  |
| --- | --- | --- | --- |

| Q2F8 | **Would you like to have other children (or a first)?** |  |  |
| --- | --- | --- | --- |
|  | Yes | 177 (87,2%) |  |
|  | No | 26 (12,8%) |  |

**1.2 You and contraception**

| Q2F9 | **Have you ever had side effects due to contraception?** |  |  |
| --- | --- | --- | --- |
|  | Yes i have | 108 (53,2%) |  |
|  | Yes my Partner has | 7 (3,4%) |  |
|  | No for none of us | 88 (43,3%) |  |

| Q2F10 | **Have you ever had a previous unwanted pregnancy while on birth control?** |  |  |
| --- | --- | --- | --- |
|  | Yes | 11 (5,4%) |  |
|  | No | 192 (94,6%) |  |

**1.3 Male contraception**

| Q2F11 | **In the list below, what type of male contraception do you know?** |  |  |
| --- | --- | --- | --- |
|  | Condom | 202 (99,5%) |  |
|  | Withdrawal | 148 (72,9%) |  |
|  | Vasectomy | 181 (89,2%) |  |
|  | Hormonal male contraception | 43 (21,2%) |  |
|  | Male contraception by hyperthermia | 20 (9,9%) |  |
|  | None | 1 (0,5%) |  |
|  | Others (please specify) | 6 (3%) |  |

| Q2M12 | **Would you agree to use a male contraception in your relationship?** |  |  |
| --- | --- | --- | --- |
|  | Oui | 150 (73,9%) |  |
|  | Non | 53 (26,1%) |  |

| Q2F13 | **If you answered YES ( question 16 ), what is your main reason ? (One answer possible)** | N=150 | 53 |
| --- | --- | --- | --- |
|  | To share contraceptive responsability | 90 (60 %) | 53 |
|  | To have an extra safety in order to avoid pregnancy | 17 (11,3%) | 53 |
|  | To avoid side effects due to female contraception | 41 (27,3%) | 53 |
|  | Prevent my partner from having a child with another partner | 0,0% | 53 |
|  | Other ( please specify ) | 2 (1,3%) | 53 |

| Q2F14 | **If you answered No (question 16), what is your main reason? ( One answer possible)** | N=53 | 149 |
| --- | --- | --- | --- |
|  | I don’t trust my partner | 15 (28,3%) | 149 |
|  | Contraception belongs to women | 4 (7,5%) | 149 |
|  | It makes my partner less virile | 6 (11,3%) | 149 |
|  | Not interested at all | 12 (22,6%) | 149 |
|  | Other (precise) | 16 (30,2%) | 149 |

| Q2F15 | **Have you ever heard of thermal male contraception?** |  |  |
| --- | --- | --- | --- |
|  | Yes | 41 (20,2%) |  |
|  | No | 162 (79,8%) |  |

Please read this short following information about the male contraception by hyperthermia and answer to question 16 to question 33.

**TMC INFORMATION Annex 2**

| Q2F16 | **Which type of male contraception would you be willing to use? (Only one answer)** | |  |
| --- | --- | --- | --- |
|  | Condom | 130 (64%) |  |
|  | Withdrawal | 10 (4,9%) |  |
|  | Vasectomy | 4 (2%) |  |
|  | Hormonal Male contraception | 16 (7,9%) |  |
|  | Male contraception by hyperthermia | 27 (13,3%) |  |
|  | None | 15 (7,4%) |  |
|  | Other (precise) | 1 (0,5%) |  |

| Q2F17 | **As to male contraception by hyperthermia, what would the pros (advantages) be? ( several answer possible)** | |  |
| --- | --- | --- | --- |
|  | Environmental | 106 (52,2%) |  |
|  | Inexpensive | 99 (48,8%) |  |
|  | No adverse effect | 108 (53,2%) |  |
|  | Efficient | 29 (14,3%) |  |
|  | Non-hormonal | 131 (64,5%) |  |
|  | Natural method | 124 (61,1%) |  |
|  | Reversible | 136 (67%) |  |
|  | None | 6 (3%) |  |
|  | Other (please specify) | 1 (0,5%) |  |

| Q2F18 | **As to male contraception by hyperthermia, what would the cons (disadvantages) be? ( several answers possible)** |  |  |
| --- | --- | --- | --- |
|  | Delayed effectiveness | 115 (56,7%) |  |
|  | Delayed reversibility | 88 (43,3%) |  |
|  | Time required for wear ( 15h per day ) | 151 (74,4%) |  |
|  | Aesthetic apperance (embarassment) | 93 (45,8%) |  |
|  | Uncomfortable | 88 (43,3%) |  |
|  | Must be wom without fail | 120 (59,1%) |  |
|  | Loss of confidence | 124 (61,1%) |  |
|  | Damage to virility | 44 (21,7%) |  |
|  | STIs risks | 6 (3%) |  |
|  | Others (please specifify) | 6 (3%) |  |

| Q2F19 | **Would you accept if your partner tried that type of male contraception?** |  |  |
| --- | --- | --- | --- |
|  | I would totally accept | 23 (11,3%) |  |
|  | I would generally accept | 68 (33,5%) |  |
|  | I would generally not accept | 83 (40,9%) |  |
|  | Not at All | 29 (14,3%) |  |

| Q2F20 | **Would you like a larger variety of**  **choice in male contraception?** |  |  |
| --- | --- | --- | --- |
|  | Yes | 182 (89,7%) |  |
|  | No | 21 (10,3%) |  |

- 1. **You, as a prescriber of contraception**

| Q2F21 | **Are you often asked to prescribe contraception?** |  |  |
| --- | --- | --- | --- |
|  | Very often | 36 (17,7%) |  |
|  | Often | 80 (39,4%) |  |
|  | Rarely | 47 (23,2%) |  |
|  | Very rarely | 14 (6,9%) |  |
|  | Never | 26 (12,8%) |  |

| Q2F22 | **Do you ever feel powerless as regards current means of contraception?** |  |  |
| --- | --- | --- | --- |
|  | Yes | 110 (54,2%) |  |
|  | No | 93 (45,8%) |  |

| Q2F23 | **How often do you propose male contraception?** |  |  |
| --- | --- | --- | --- |
|  | Very often | 5 (2,5%) |  |
|  | Often | 31 (15,6%) |  |
|  | Rarely | 54 (26,6%) |  |
|  | Very rarely | 49 (24,1%) |  |
|  | Never | 64 (31,5%) |  |

| Q2F24 | **Have you ever proposed other types of male contraception than condoms?** |  |  |
| --- | --- | --- | --- |
|  | Very often | 0,0% |  |
|  | Often | 3 (1,5%) |  |
|  | Rarely | 38 (18,7%) |  |
|  | Very rarely | 57 (28,1%) |  |
|  | Never | 105 (51,7%) |  |

| Q2F25 | **If you answered yes, which one(s)?** |  |  |
| --- | --- | --- | --- |
|  | Vasectomy | 78 (38,4%) |  |
|  | Hormonal male contraception | 3 (1,5%) |  |
|  | Thermal male contraception | 9 (4,4%) |  |
|  | None | 118 (58,1%) |  |
|  | Other (please specify) | 5 (2,5%) |  |

| Q2F26 a | **If you do not propose vasectomy, why? (Several answers possible )** | n=125 |  |
| --- | --- | --- | --- |
|  | I don’t know about it | 18 (15,8%) |  |
|  | I have no confidence in it | 2 (1,8%) |  |
|  | I don’t have any qualification to prescribe it | 39 (34,2%) |  |
|  | I don’t know any appropriate colleague I could liaise with | 29 (25,4%) |  |
|  | I’m concerned about side effects | 11 (9,6%) |  |
|  | It never occurs to me | 45 (39,5%) |  |
|  | Other (please specify) | 18 (15,8%) |  |

| Q2F26 b | **If you do not propose hormonal male contraception, why? (Several answers possible)** | n=194 |  |
| --- | --- | --- | --- |
|  | I don’t know about it | 132 (71%) |  |
|  | I have no confidence in it | 45 (2,2%) |  |
|  | I don’t have any qualification to prescribe it | 60 (32,3%) |  |
|  | I don’t know any appropriate colleague I could liaise with | 36 (19,4%) |  |
|  | I’m concerned about side effects | 37 (19,9%) |  |
|  | It never occurs to me | 27 (14,5%) |  |
|  | Other (please specify) | 9 (4,8%) |  |

| Q2F26 c | **If you do not propose thermal male contraception, why? (several answers possible)** | n= 200 |  |
| --- | --- | --- | --- |
|  | I don’t know about it | 156 (81,2%) |  |
|  | I have no confidence in it | 30 (15,6%) |  |
|  | I don’t have any qualification to prescribe it | 26 (13,5%) |  |
|  | I don’t know any appropriate colleague I could liaise with | 25 (13%) |  |
|  | I’m concerned about side effects | 20 (10,5%) |  |
|  | It never occurs to me | 21 (10,9%) |  |
|  | Other (please specify) | 3 (1,6%) |  |

| Q2F27 | **Would you be willing to recommend male contraception by hyperthermia?** |  |  |
| --- | --- | --- | --- |
|  | Yes | 127 (62,6%) |  |
|  | No | 76 (37,4%) |  |

| Q2F28 | **As to male contraception by hyperthermia, what would the pros (advantages) be FOR YOUR PATIENTS? ( several answer possible)** | |  |
| --- | --- | --- | --- |
|  | Environmental | 102 (50,2%) |  |
|  | Inexpensive | 124 (61,1%) |  |
|  | No adverse effect | 113 (55,7%) |  |
|  | Efficient | 27 (13,3%) |  |
|  | Non-hormonal | 150 (73,9%) |  |
|  | Natural method | 145 (71,4%) |  |
|  | Reversible | 133 (65,5%) |  |
|  | None | 3 (1,5%) |  |
|  | Other (please specify) | 3 (1,5%) |  |

| Q2F29 | **As to male contraception by hyperthermia, what would the cons (disadvantages) be FOR YOUR PATIENTS? ( several answers possible)** | |  |
| --- | --- | --- | --- |
|  | Delayed effectiveness | 130 (64%) |  |
|  | Delayed reversibility | 100 (49,3%) |  |
|  | Time required for wear ( 15h per day ) | 159 (78,3%) |  |
|  | Aesthetic apperance (embarassment) | 119 (58,6%) |  |
|  | Uncomfortable | 91 (44,8%) |  |
|  | Must be wom without fail | 140 (69%) |  |
|  | Loss of confidence | 112 (55,2%) |  |
|  | Damage to virility | 68 (33,5%) |  |
|  | STIs risks | 95 (46,8%) |  |
|  | Others (please specifify) | 0,0% |  |

| Q2F30 | **Would you like to have more information about male contraception in general?** |  |  |
| --- | --- | --- | --- |
|  | Yes | 195 (96,1%) |  |
|  | No | 8 (3,9%) |  |

| Q2F31 | **Would you be interested to participate to a training course about male contraception?** |  |  |
| --- | --- | --- | --- |
|  | Yes | 173 (85,2%) |  |
|  | No | 30 (14,8%) |  |

**Thank you for your collaboration!**
